# Influence of Various Heat Treatments on Microstructures and Mechanical Properties of GH4099 Superalloy Produced by Laser Powder Bed Fusion

**DOI:** 10.3390/ma17051084

**Published:** 2024-02-27

**Authors:** Jiahao Liu, Yonghui Wang, Wenqian Guo, Linshan Wang, Shaoming Zhang, Qiang Hu

**Affiliations:** 1 Industrial Research Institute for Metal Powder Material, China GRINM Group Co., Ltd., Beijing 101407, China; lewin068@163.com (J.L.);; 2 General Research Institute for Nonferrous Metals, Beijing 100088, China; 3 GRINM Additive Manufacturing Technology Co., Ltd., Beijing 101407, China

**Keywords:** nickel-based superalloy, laser powder bed fusion, heat treatment, microstructure, tensile properties

## Abstract

The microstructures and mechanical properties of a γ′-strengthened nickel-based superalloy, GH4099, produced by laser powder bed fusion, at room temperature and 900 °C are investigated, followed by three various heat treatments. The as-built (AB) alloy consists of cellular/dendrite substructures within columnar grains aligning in <100> crystal orientation. No γ′ phase is observed in the AB sample due to the relatively low content of Al +Ti. Following the standard solid solution treatment, the molten pool boundaries and cellular/dendrite substructures disappear, whilst the columnar grains remain. The transformation of columnar grains to equiaxed grains occurs through the primary solid solution treatment due to the recovery and recrystallization process. After aging at 850 °C for 480 min, the carbides in the three samples distributed at grain boundaries and within grains and the spherical γ′ phase whose size is about 43 nm ± 16 nm develop in the standard solid solution + aging and primary solid solution + aging samples (SA and PA samples) while the bimodal size of cubic (181 nm ± 85 nm) and spherical (43 nm ± 16 nm) γ′ precipitates is presented in the primary solid solution + secondary solid solution + aging sample (PSA samples). The uniaxial tensile tests are carried out at room temperature (RT) and 900 °C. The AB sample has the best RT ductility (~51% of elongation and ~67% of area reduction). Following the three heat treatments, the samples all acquire excellent RT tensile properties (>750 MPa of yield strengths and >32% of elongations). However, clear ductility dips and intergranular fracture modes occur during the 900 °C tensile tests, which could be related to carbide distribution and a change in the deformation mechanism.

## 1. Introduction

Additive Manufacturing (AM) is becoming popular and has been adopted by different industries; it is based on a layer-by-layer additive process controlled by highly automated computer model software. It makes use of powder-based and wire-based feedstock materials to form components that experience rapid solidification and a thermal cycle, with different heating sources including lasers, electron beams, and plasma arcs [1,2,3]. In particular, laser powder bed fusion (LPBF) is a typical AM process used to build parts by spreading the sequential layers with a thickness of 20 µm~50 µm of metal feedstock powder melted by a high-velocity scanning laser. A pronounced feature of LPBF is that the cooling rate of the melt pool reaches 10^6^ K/s~10^8^ K/s, which substantially limits elemental segregation in the range of a few micrometers. However, cracking, micro-defects and anisotropy of its properties still appear in the LPBF alloys leading to premature failure, residual stress and heterogeneous microstructures, which presents a huge challenge toinvestigation and application [4,5,6,7]. In a word, more creative technology can not only provide more room for flexibility in manufacturing parts but can also significantly reduce processing time and cost. Meanwhile, the trend of lightweight and high strength design has gone viral, resulting in increased demand for geometrically complex shapes and internal structures in many key components [8,9].

Nickel-based superalloys have been the primary candidate class of alloys for high-temperature applications in the aerospace and nuclear power industries because of their excellent resistance to creep, adequate corrosion resistance and the ability to tune their microstructure for desirable mechanical properties. At present, a great number of superalloys available for LPBF have been studied such as Inconel 718, Inconel 625, Haynes 230, Inconel 738LC, Rene 41, CMSX-4 [10,11,12,13,14,15,16,17], etc. Superalloys in the LPBF or heat-treated condition have demonstrated comparable or improved strength in comparison to the conventionally manufactured counterparts, but usually show a dip in ductility, as reported in the previous works [18,19,20]. Shaikh et al. [18] determined the anisotropy of the LPBF superalloy by comparing the tensile elongation of vertically and horizontally heat-treated Haynes 282 alloy at room temperature and 800 °C, which varied by approximately 50%. These are due to the difference in the grain boundary area and susceptibility to cavitation between horizontal and vertical directions when applied to a tensile load. Also, the elongation of LPBF materials at 800 °C was likewise below the level (~28%) of elongation at 816 °C for the reference plate Haynes 282 alloy, which was related to the configuration of the γ′ phase and grain boundary carbide. Kuo et al. [19] found that the as-built and direct-aged Inconel 718 had a much lower creep rupture life because of the stress concentration in the brittle δ phase and the movement of high-density dislocations which was confined by substructures. Kreitcberg et al. [20] suggested that the orientation of the plate δ phase and the size of M_6_C at the grain boundary following various heat treatments in the LPBF IN625 led to differences in the tensile properties. In addition, the formation and coarsening of M_6_C carbides at the grain boundaries during tensile testing and the overall length of the columnar grain boundaries were responsible for the decline in elongation at 750 °C compared with annealing wrought IN625. As a result, the phase evolutions of LPBF superalloys are prominently distinguished from the conventionally manufactured counterparts. More studies are needed to guarantee the reliability of an additive-manufactured superalloy for high-temperature applications.

Recently, attention has been drawn to the LPBF GH4099 superalloy for aerospace applications. It is mainly utilized in aerospace engine combustion chambers and empennages of missiles due to its excellent microstructural stability at ~900 °C, high corrosion resistance [21,22] and weldability [23]. The GH4099 superalloy is a precipitation-strengthened nickel-based superalloy with the addition of ~3.5 wt.% Al + Ti to form γ′-Ni_3_(Al, Ti) phase. Also, ~17 wt.% Co + W + Mo and >18 wt.% Cr as solid solution elements, along with minor elements such as B and Mg as grain boundary-strengthening elements are added to the alloy. To date, several studies on the additive-manufactured GH4099 superalloy have been carried out. Hu et al. [24] displayed the microstructure of GH4099 built by directed energy deposition (DED), consisting of epitaxial growth columnar grains along the building direction, with the γ/γ′ eutectic phase dispersing in the inter-dendritic regions. They identified that the microstructure was changed to equiaxed grains after standard solid solution treatment due to recrystallization. Li et al. [25] found the gradient microstructures of the GH4099 produced by DED, in which the MC carbide formed in the top and bottom regions of the alloy whilst the M_23_C_6_ carbide formed in the middle region. Following the heat treatment, the top region showed a 79.99% decrease in average grain size, while the middle and bottom regions showed a slight increase in average grain size. This was due to the presence of recrystallization grains during the as-deposited process and eventually grain growth after heat treatment in the middle and bottom regions. However, the top region was the final solidification region without continuous heat input leading to a large columnar grain. The fine recrystallization grains, in turn, were formed after heat treatment. This led to the microhardness gradient among the three regions. Chang et al. [26,27] prepared a GH4099 superalloy using LPBF. The microstructure and strengthening mechanisms at room temperature were characterized and the strengthening contributions under the conditions of solid solution treatment and aging treatment were calculated. Zhang et al. [28] explored the anisotropy of tensile properties at room temperature and 900 °C and found that it was related to the difference in grain morphology arising from the different solid solution temperature. The presence of mechanical anisotropy at room temperature was highly related to the difference in grain boundary density. At 900 °C, more stacking faults and deformation twins could accommodate higher plastic deformation when the tensile load was applied along the building direction. In addition, Wang et al. [29] made a comparison between GH4099 alloys fabricated by Electron Beam Melting, which were prepared using VIGA powder and PREP powder, respectively. Lu et al. [30] analyzed the influence of microstructural evolution on the electrochemical corrosion performance of SLM GH4099 superalloy. Lu et al. [31] investigated the residual stress evolution of the bridge parts for LPBF GH4099 and its annealing heat treatment counterparts.

Based on the above, in this study, a GH4099 superalloy was produced by LPBF technology, and it is of paramount importance to design novel heat treatments and investigate the influences of various microstructures on the tensile properties of an LPBF GH4099 alloy at room temperature and 900 °C. This research will provide theoretical guidance for the engineering application of LPBF high-performance GH4099 superalloy components.

## 2. Materials and Methods

### 2.1. Materials and Apparatus

**Figure 1:**
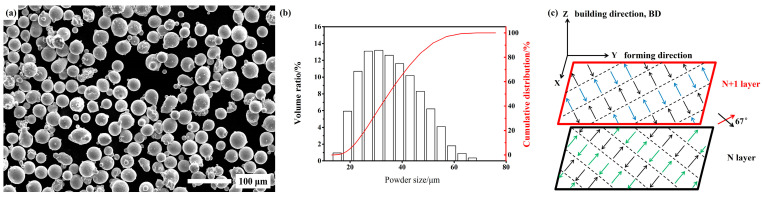
(**a**) The morphology of VIGA GH4099 powder; (**b**) Powder size distribution; (**c**) Schematic diagram of forming strategy.

The vacuum induction-melting gas atomization (VIGA) GH4099 alloy powder with an average particle size of 32.80 µm was used as the raw material. The powder morphology and size distribution are shown in Figure 1a,b. The main chemical compositions of the powder are given in Table 1. The LPBF GH4099 superalloy (as-built sample, designated AB sample) was produced using an ASA-260M printing machine designed by China Aerospace Science and Industry Co., Ltd. (Beijing, China), with the following process parameters: laser power of 280 W, scanning speed of 1200 mm/s, hatch spacing of 0.10 mm and layer thickness of 0.04 mm. The scanning direction between adjacent layers was rotated by 67° and in the “zigzag” path to reduce defects and remove the anisotropy in XOY planes. The protective gas was argon during the whole process. The forming strategy is shown schematically in Figure 1c.

The cubic samples with dimensions of 12 × 12 × 12 mm^3^ were manufactured to observe the microstructures. Cylindrical bar samples (φ10 mm × 68 mm) were built in the vertical direction (BD parallel to the tensile direction). After manufacturing, the samples were cut from the stainless-steel building plate using an electrical discharge machine to carry out the subsequent heat treatments. The different heat treatments, summarized in Table 2, were then applied in a tube furnace (GSL-1750X, Hefei kejing materials technology Co., Ltd., Hefei, China) in an argon atmosphere.

### 2.2. Microstructural Analysis

The YOZ planes (the building direction) of as-built samples and heat-treated samples were first ground with 600, 1200, 2000, and 3000 mesh SiC papers and then polished with 1 μm diamond solution and 50 nm SiO_2_ suspension. The samples were chemically etched to observe the grain structure and carbide phase using the mixture of 5 g CuCl_2_ + 100 mL HCl + 100 mL C_2_H_5_OH and were electrolytically etched to observe the γ′ phase using the mixture of 15 g CrO_3_ + 10 mL H_2_SO_4_ + 170 mL H_3_PO_4_ for 5 s~8 s at 5 V direct current (DC) power. We used optical microscopy (OM, Zeiss Axio Vert. A1, Carl Zeiss AG, Oberkochen, Germany), scanning electron microscopy (SEM, JSM-7900F, JEOL, Tokyo, Japan) equipped with an Energy Dispersive Spectrometer (EDS) and transmission electron microscopy (TEM, Talos F200X G2, Thermo Fisher Scientific, Massachusetts, The U.S. America) to observe the microstructures, and measured the size of the structures using ImageJ software. We used EBSD to analyze the crystallographic orientations and recrystallization of samples after electrolytically polishing in the mixture of 20 vol% H_2_SO_4_ + 80 vol% CH_3_OH for 15 s at 15 V DC power. At least 2 mapping areas of 1000 × 1000 μm^2^ were indexed with a step size of 2 μm.

### 2.3. Tensile Properties Characteristics

The uniaxial tensile test at room temperature was measured using an XN-298 5928 (Shanghai XingNiu Instrument Co., Ltd., Shanghai City, China) equipped with an extensometer (gauge length is 25 mm). The strain rate of the room-temperature tensile test was 1 mm/min. The uniaxial tensile test at 900 °C with a strain rate of 8.7 × 10^−5^ s^−1^ and 5 × 10^−4^ s^−1^ before and after the yield stage was carried out using an AG-250KNIC mechanical testing machine (Shimadzu, Kyoto, Japan). The room temperature uniaxial tensile test bars with an original gauge of φ4 mm × 30 mm and the 900 °C uniaxial tensile test bars with an original gauge of φ5 mm × 25 mm were carefully machined. All reported tensile test results are averages of 3 samples per treating condition.

## 3. Results

### 3.1. Microstructural Characteristics

#### 3.1.1. Solid Solution Heat Treatment

The microstructures of the as-built sample are displayed by Figure S1 in the Supplementary Materials. Briefly, the alloy is mainly composed of cellular/dendrite substructures within elongated columnar grains along the building direction (vertical section) due to the rapid cooling that grows along <100> crystal orientation. Solid solution heat treatment is a crucial step for fine-tuning the grain size, morphology and distribution of the carbide phase to further control mechanical properties. It also eliminates residual stress and reduces the anisotropy of the AB sample.

**Figure 2:**
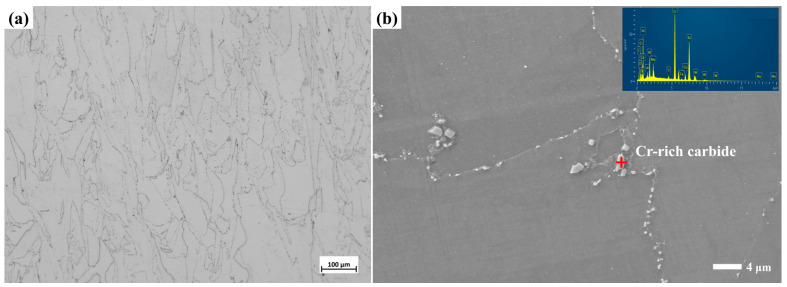

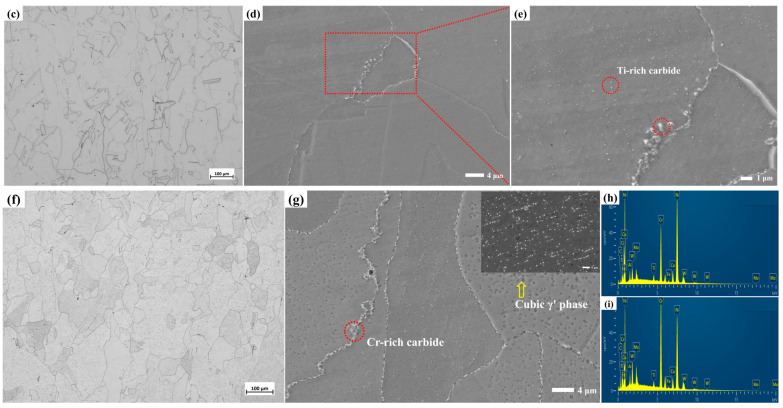
Microstructures of YOZ plane showing grain morphology of LPBF GH4099 after various solid solution treatments (**a**,**b**) SA sample; (**c**–**e**) PA sample; (**f**,**g**) PSA sample, along with (**h**) the EDS of matrix and (**i**) the EDS of carbide.

**Figure 3:**
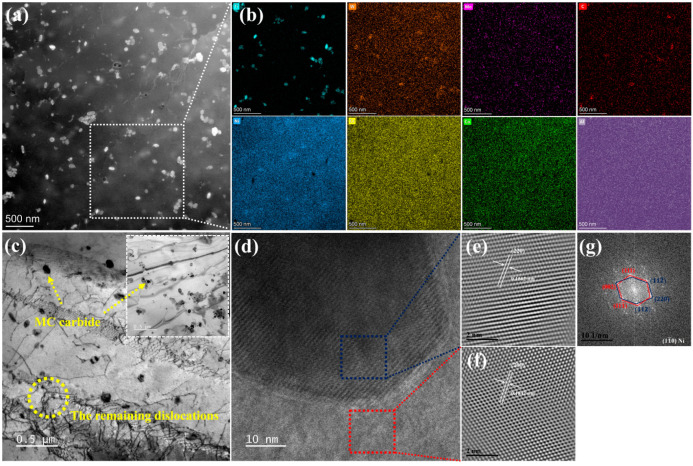
The TEM results of the PA sample under primary solid solution condition (**a**,**b**) STEM image and TEM-EDS results; (**c**) bright-field images; (**d**) high-resolution image of carbide and matrix; (**e**,**f**) inverse FFT image of MC carbide and matrix; (**g**) SAED of interface between carbide and matrix.

Optical micrographs and SEM images of the microstructures of YOZ planes of the SA, PA and PSA samples are exhibited in Figure 2. The microstructures of the SA sample are noticeably different from those of the PA and PSA ones, in which recrystallization seldom takes place. The molten pool boundaries have completely disappeared and the elongated columnar grains along the building direction are mainly presented in the alloy. In the high-magnification SEM image, discrete carbide particles, ranging in size from ~0.2 µm to ~2 µm, are dispersed at grain boundaries. Also among columnar grains, there are tiny carbide particles that remain in between the original cellular structure. This is an indication that most of the carbides may disintegrate after heating at 1140 °C for 120 min. Then, a significant quantity of equiaxed grains with annealing twins and columnar grains coexist in the PA sample, which demonstrates that the alloy undergoes a recrystallization process. The TEM bright-field image in Figure 3c shows that there are the remaining dislocations along the original substructure which the density decreases from 6 ± 0.4 × 10^11^ m^−2^ in the AB sample to 8 ± 1.3 × 10^8^ m^−2^. With a duration time at higher solid solution temperature, the very fine primary carbide particles grow and distribute uniformly throughout the matrix. The radius of carbide particles affects the stability. The smaller the radius, the higher the Gibbs free energy is [32]. Therefore, parts of the tiny particles dissolve and the rest merge and grow due to falling Gibbs free energy to obtain a stable state. Actually, there is a decrease in carbide content and a minor rise in size. Compared with that of the PA sample, the PSA sample exhibits a more pronounced equiaxial conversion propensity. The small and discontinuous carbide particles distribute at grain boundaries and within grains. Moreover, there are a host of tiny carbide particles that originate from the transformation of the carbide phase that forms in the primary solid solution, accompanied by the sparse cubic γ′ precipitates that disperse within the grains at a size of approximately 158 nm ± 48 nm. As shown in Figure 2g inset, the cubic γ′ phase within grains is regularly arranged along the direction of the initial dendritic structures which is mostly dependent on the various grain orientations. The interval of each row of γ′ phase is nearly equal to the width of the original dendritic structures. The volume fraction of the cubic γ′ phase is much lower (volume fraction of ~3.7 vol.%), where 1010 °C is supposed to be close to the complete dissolving temperature of the γ′ phase of the GH4099 superalloy.

SEM-EDS and TEM analysis are used to identify the different carbide phase types. As known from the EDS result of Figure 2b inset, the Cr-rich phase with W, Mo and C forms in the alloy are subjected to a standard solid solution, in which they are determined as M_23_C_6_ carbides [26,33]. The round and fine MC particle is featured by TEM results, as displayed in Figure 3. In addition to the obvious enrichment of Ti in the particles, a minor amount of W, Mo, and C also segregate. As known from the TEM-EDS mapping results of Figure S3, Ti mainly segregates at the inter-dendritic (IR) region in the AB sample. Therefore, it can be seen that the MC particles grow in the previous IR region. The size of MC particles increases to 60 nm~150 nm, compared with that of the AB sample, which indicates that a higher solution temperature is conducive to the aggregation and growth of MC carbides, and the Ti-rich MC carbide has good thermal stability [34]. The high-resolution picture and SAED characteristics of MC carbide show that it shares a comparable FCC crystal structure with the matrix. It has a semi-congruent relationship with the matrix, with the (220)_MC_ crystal plane nearly parallel to the (200)_γ_ crystal plane. After secondary solid solution treatment, the carbides enriched with Cr, W, and Mo are again generated, which are consistent with the chemical composition of the M_23_C_6_-type carbides. This has been identified in other works [35,36] and they are thus termed as (Cr, W, Mo)_23_C_6_. The phase transformation from MC to M_23_C_6_ and grain boundary immigration happens leading to the formation of a discrete grain boundary and interior M_23_C_6_ particles during the secondary solid solution process.

**Figure 4:**
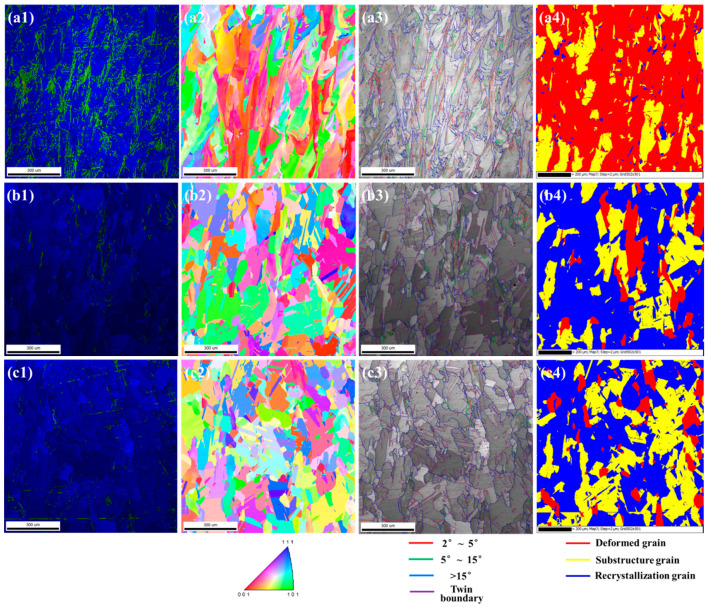
The EBSD results of three alloys (**a1**–**a4**) SA sample; (**b1**–**b4**) PA sample; (**c1**–**c4**) PSA sample (the first column (from left) shows KAM figures. The second column shows inverse pole figures. The third column shows grain misorientation mappings. The fourth column shows recrystallization mappings).

EBSD analysis is conducted to investigate the microstructural evolution at different solid solution treatments. The grain structures have changed in comparison to that of the AB sample observed in Figure S2. Figure 4 illustrates the KAM figures, inverse pole figure (IPF), grain boundary misorientation and recrystallization mapping along the building direction of three samples. From the observation of KAM figures, there is a high level of residual stress with a magnitude of ~20.9% in the SA sample. With the increase in primary solid solution temperature and the application of secondary solid solution, the magnitudes of the residual stress of the PA sample and PSA sample gradually drop to ~2.2% and ~1.6%, respectively. The transformation from columnar grains to equiaxed grains thus occurs, and the average equivalent grain sizes are obtained based on the EBSD results, where they are 105.17 µm, 94.04 µm and 117.58 µm, respectively. In addition, the intensity of the preferred orientation of <100> weakens with the rise in temperature and steps. In response to the change in grain morphologies, the grain boundary (GB) misorientation and transformation of grain also changes, as depicted in Figure 4(a3,b3,c3). Accordingly, the statistical results are tabulated in Table 3 and Table 4. When heat-treated at 1140 °C, low-angle GBs (2°~15°, LAGBs), where the residual stress stays mostly appear within columnar grains. Then, following the primary solid solution heat treatment, LAGBs gradually disappear. This occurs as a result of high-angle grain bodies (>15°, HAGBs) migrating to accomplish further grain growth. Through the subsequent secondary heat treatment at 1010 °C for 240 min, the residual stress in the sample is further released and the recrystallization process proceeds, as displayed in Figure 4(a4,b4,c4). The accumulation of large residual stresses which is attributed to the extremely large temperature gradients and rapid cooling rates during the SLM process, as a driving force, leads to recrystallization when the thermal activation energy in the LPBF superalloy is sufficiently high, i.e., when there is a rise in the temperature of the solid solution treatment [37,38,39].

#### 3.1.2. Solid Solution and Aging Condition

**Figure 5:**
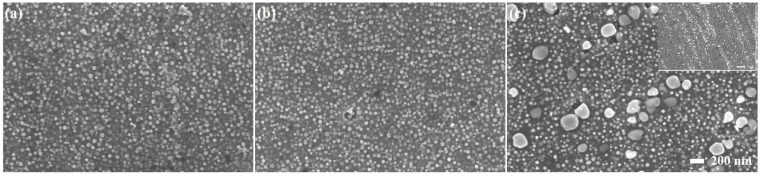
The morphologies of γ′ precipitates (**a**) SA sample, (**b**) PA sample and (**c**) PSA sample.

The configuration of the γ′ phase has a decisive influence on the mechanical properties of Ni-based superalloys [40,41,42]. In general, greater strength and lower ductility are associated with a higher volume fraction of the γ′ phase. Strength is further affected by the interaction (shearing or bypassing) between the γ′ phase and dislocations, which is dependent on the size of the γ′ phase. Figure 5a–c shows the morphologies of γ′ precipitates of the three alloys subjected to 850 °C for 480 min and then cooled in air. At a relatively high cooling rate, the size distribution of γ′ precipitates is unimodal, in which spherical γ′ precipitates uniformly distribute in the matrix and the equivalent circle diameter is approximately 43 nm ± 16 nm in the SA and PA samples. The volume fractions of spherical γ′ precipitates for these two samples are about 40.83 vol.% and 41.39 vol.%, respectively. Meanwhile, the round and cubic γ′ particles show that the PSA sample has developed a bimodal γ′ distribution. Quantitative analysis shows that the overall volume fraction of γ′ precipitates is about 41.04 vol.%, including ~14.49 vol.% (181 nm ± 85 nm) of secondary γ′ precipitates and ~26.55 vol.% (43 nm ± 16 nm) of tertiary γ′ precipitates. After the aging process, the tertiary γ′ precipitates uniformly precipitate and the earlier nucleated secondary γ′ precipitates continue to grow (3.7 vol.% to 14.49 vol.%). During the process of secondary solid solution treatment, the strengthening elements diffuse to the vicinity of vacancies and remaining dislocations to form secondary γ′ precipitates and very few tertiary γ′ precipitates begin to nucleate. 

**Figure 6:**
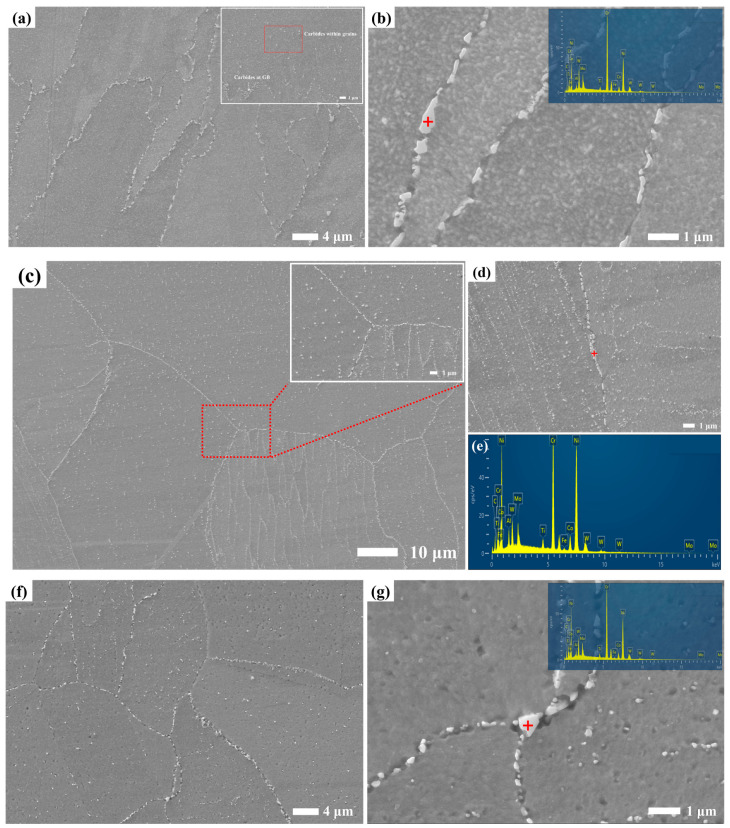
The carbide phase in the (**a**,**b**) SA sample; (**c**,**d**) PA sample, along with (**e**) the EDS of carbide; (**f**,**g**) PSA sample (the sites where are pointed by red cross symbol are M_23_C_6_ carbide particles).

It is of paramount significance in the evolution of the carbide phase in the three alloys, which directly affects the tensile properties, as exhibited in Figure 6. As seen by the red dashed line box, the irregular carbide particles in the SA sample grow discretely not only at the grain boundaries but also sparsely along the original substructure boundaries. Similarly, a large number of small carbide particles within grains are precipitated, which nearly shows the contour of the original cellular structure in the PA sample. Meanwhile, the semi-continuous carbide particles grow at the grain boundaries. A similar carbide growth is observed in [38]. Additionally, carbide formation is still seen in the PSA sample, although it does so in a different distribution than in the PA sample. The carbide particles within grains disperse randomly and uniformly and the ones at GBs are discrete. Similarly, the Cr-rich M_23_C_6_ carbide phases in three alloys are identified by SEM-EDX after the aging process, precipitated or transformed at the temperature of 750 °C~1150 °C [27,28,43,44,45]. The size changes in carbide particles at GBs and the grain interior under the various settings and samples are measured and the transformation and evolution of the carbide phase will be discussed in Section 4.1.

### 3.2. Mechanical Properties

**Figure 7:**
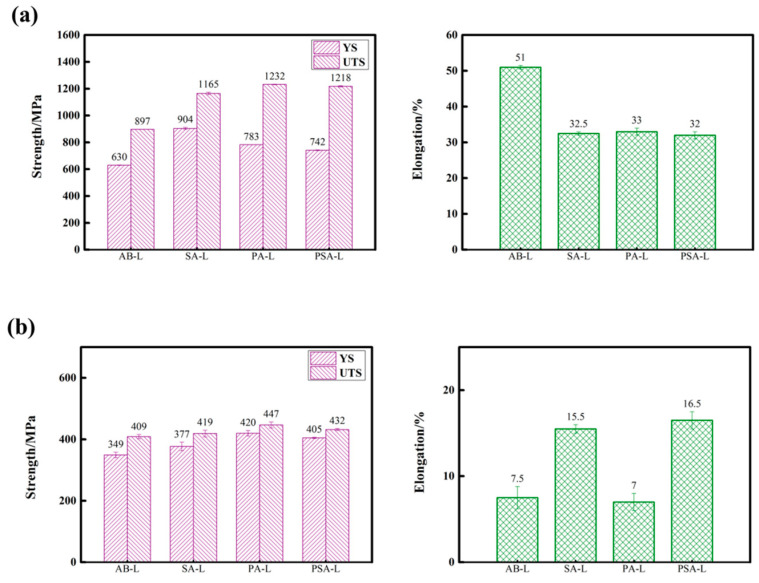
Uniaxial tensile properties of LPBF GH4099 superalloy in as-built and heat-treated conditions at (**a**) room temperature and (**b**) 900 °C (YS: yield strength, UTS: ultimate tensile strength, EL: elongation).

The histograms of uniaxial tensile properties of LPBF GH4099 superalloy subjected to three heat treatment conditions at room temperature and 900 °C are presented in Figure 7. At room temperature, the as-built sample shows remarkable ductility (~51% of elongation and ~67% of area reduction). Based on the previous results, there is no carbide and γ′ phase in the AB sample. Therefore, its tensile properties are generated by mainly grain boundary strengthening (very fine cellular substructures) and dislocation strengthening (high-density entangled dislocations) [46,47]. It is also clearly shown that the heat treatment conditions play a significant role in the tensile properties arising from the occurrence of γ′ precipitates and carbide phase.

Among three heat-treated samples, as known by the KAM values, the proportions of residual stress in the SA, PA, and PSA samples decrease and the entangled dislocations slip paths of them increase. This means that the applied stress on the SA sample required for the dislocation slip accordingly increases. The SA sample therefore has the highest yield strength. However, its earlier failure and lowest tensile strength are caused by the relatively large size of carbide particles at grain boundaries and the concentrated stress induced by dislocations entangling. The PA sample has a relatively higher yield strength than the PSA sample, despite having similar tertiary γ′ precipitates and recrystallization microstructures. This is due to the PSA sample having coarsened secondary γ′ precipitates and the PA sample having a higher carbide content.

The tensile properties clearly decrease at 900 °C, particularly the ductility. Among the three heat-treated samples, the PA sample has the highest strength (YS is 420 MPa and UTS is 447 MPa) and the lowest elongation (~7%). It is worth noting that the tensile properties of the PA and PSA sample seem to be quite comparable at room temperature but the elongation of the PSA sample is higher by more than one-fold than that of the PA sample at elevated temperature. The stress-strain relationship is influenced by several factors in Ni-based superalloys such as grain size, grain boundary situation, the configuration of γ′ phase, homogeneity or chemical composition. These striking differences in tensile properties will be discussed in Section 4.2.

## 4. Discussion

### 4.1. The Evolution of Carbide Phase

The carbide phase has a striking effect on the mechanical properties of superalloys depending on their morphology, size and distribution. Fine and discontinuous carbide particles are capable of impeding grain boundary slip and dislocation motion, which are believed to be the primary factors influencing an alloy’s tensile properties. On the contrary, coarse or continuous carbides are detrimental, where they are prone to enhance cracking susceptibility [48]. The work indicates that the M_23_C_6_ carbide is almost desirable to favor the high-temperature tensile properties and creep life [49]. Therefore, it is critical to optimize the heat treatment strategies to control the phase stability and morphological evolution of different carbides.

**Figure 8:**
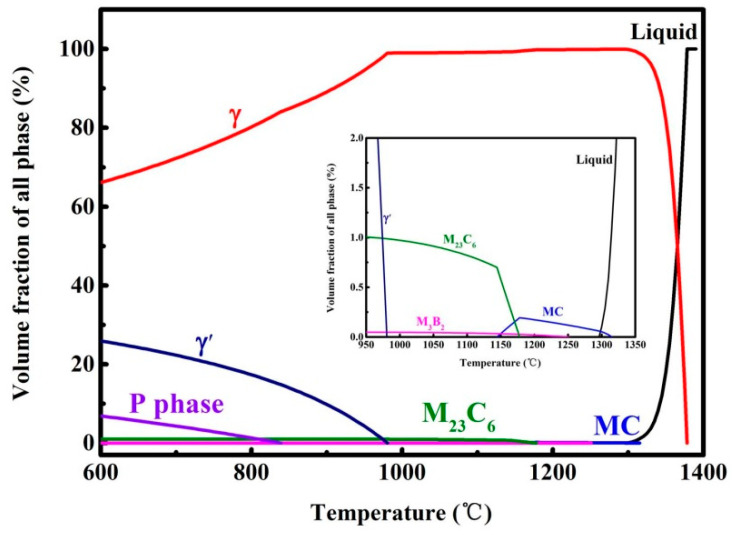
The equilibrium phase diagram of GH4099 using the JMatPro 7.0 software.

Figure 8 shows the temperature-dependent equilibrium phase diagram calculated by JMatPro 7.0 software based on the given chemical composition in Table 1. It can be found that the types of the phase predicted consist of γ, γ′, M_23_C_6_, MC, M_3_B_2_, and TCP phase under equilibrium conditions. The B-containing phase can be neglected due to very low content (<40 ppm). The TCP phase, and P phase, which is regarded as a harmful phase, are not found in the GH4099 superalloy. Thus, the phase transformation in the heat-treated GH4099 superalloy mainly refers to MC, M_23_C_6_ and γ′.

Based on the calculated equilibrium phase diagram, the MC carbide firstly forms during solidification and disappears at a temperature of about 1145 °C. Subsequently, the M_23_C_6_ carbide starts to form when the temperature drops to about 1180 °C. Finally, the γ′ phase begins to precipitate till ~980 °C. Therefore, the MC carbide and M_23_C_6_ carbide are considered as the primary carbide and secondary carbides, respectively. The elements, such as Ti and W, are both strong formers of carbides. The formation temperature of the WC carbide in the alloy is around 2500 °C which is far greater than the temperature of the liquid superalloy. Therefore, the TiC-type carbide initially forms during the solidification and parts of W and Mo may replace the Ti atoms [50]. Cr is a weaker carbide former than Ti and W, despite the fact that Cr atoms dissolve uniformly in the matrix. Also, there is not enough time for the Cr-C carbide to develop due to its rapid cooling rate. According to [51], there is a carbide transformation formula as follows:

(1) $$\mathrm{MC}+\gamma \to M_{23}C_{6}+\gamma^{\prime }$$

**Figure 9:**
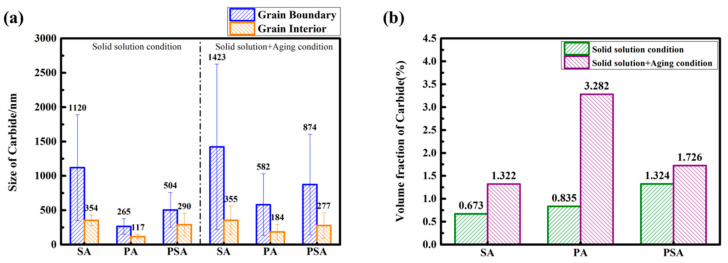
The (**a**) size and (**b**) volume fraction change in carbide in the SA sample, PA sample and PSA sample at solid solution condition and solid solution + aging condition.

The M_23_C_6_ carbide is introduced by the major pathway above. Figure 9 shows the size and volume fraction change in the carbide in the three samples at different heat treatment conditions. It is pronounced that the M_23_C_6_ carbide inclines to precipitate at the grain boundary, which is attributed to the higher energy barrier between the γ/M_23_C_6_ interface on the precipitation, leading to the preferable nucleation and growth at the kinetically feasible grain boundaries [52]. In terms of the theories above, it can be inferred that M_23_C_6_ carbide particles form in small accounts at grain boundaries and within grains following the standard solid solution due to the temperature of 1140 °C which is near their dissolving point. Then, Ti-rich MC particles form and grow due to the micro-segregation of Ti at cellular boundaries when the solid solution temperature rises to 1205 °C. Compared to the SA sample, the carbon atoms in the matrix reach super-saturation of concentration and have the appropriate thermodynamic and dynamic conditions for nucleation and growth. Therefore, carbon atoms can strongly diffuse from the grain interior to the boundaries to form particle-like carbides and grow into the semi-continuous rod-like morphology during aging at 850 °C. A similar phenomenon can be observed in [53]. The secondary solid solution treatment, called “carbide stability treatment”, is to encourage the growth of discontinuous carbide particles at grain boundaries and to cause a relatively small increase in size and volume fraction following the aging process [54].

### 4.2. The Differences of Mechanical Performance

**Figure 10:**
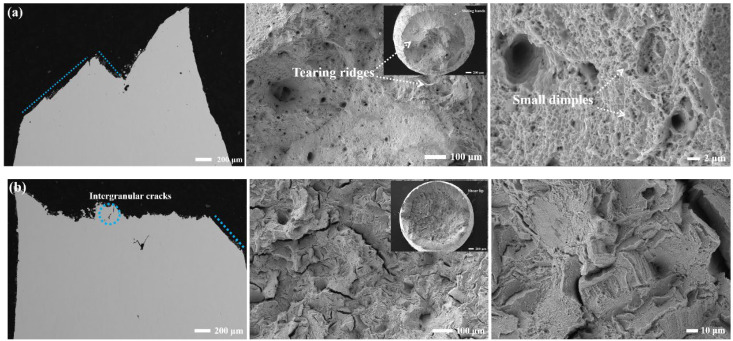

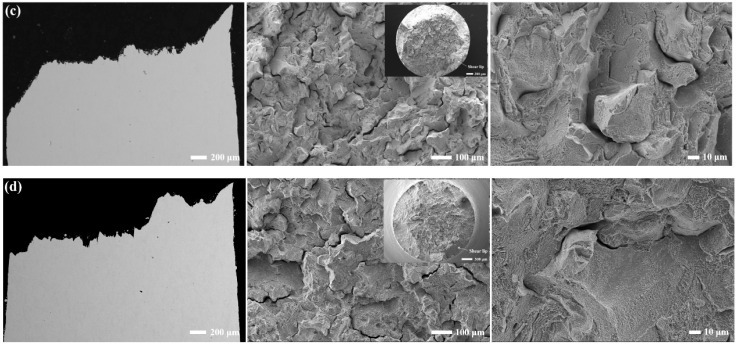
The fracture surface morphologies of (**a**) AB sample; (**b**) SA sample; (**c**) PA sample and (**d**) PSA sample tensile tested at room temperature.

Figure 10a shows the fracture surfaces of the as-built sample tensile tested at room temperature. A large number of slip bands can be evidently seen in the tensile bar body and tearing ridges are seen from the inset. There are also lots of pores and very fine dimples on the fracture surface. The typical dimple and tearing feature on the fracture surface can be regarded as a ductile mode. These observations correlate well with the high elongation (~51%) and reduction in area (~67%) measured during tensile testing at room temperature.

The fracture modes of three heat-treated samples are changed due to precipitations of carbide and γ′ phase. Figure 10b–d shows that the fracture surface contains many small intergranular cracks. Fine dimples and tearing ridges are still observed, along with some parallel slip ribbons. Some fracture stairs indicate the occurrence of intragranular fractures. The back-scattering electron (BSE) cross-section morphologies show that the cracks are propagated both through grains and along grain boundaries. In addition, the absence of any secondary cracks beneath the fracture surface can be found. The fracture modes of the PA and PSA samples are similar, indicating a combination of intergranular and intragranular fractures. Also, the morphology of rock-candy-like grains is observed due to the transformation from columnar grains to equiaxed grains.

**Figure 11:**
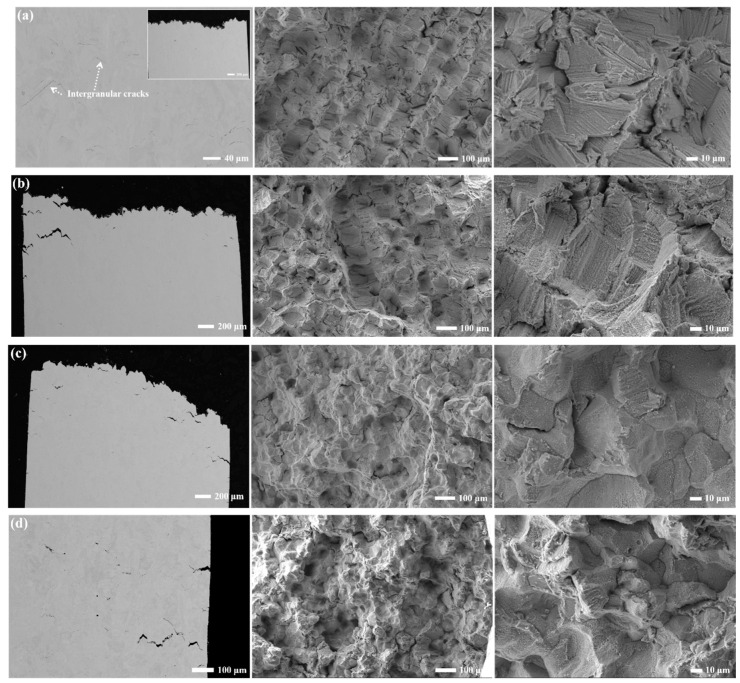
The fracture surface morphologies of (**a**) AB sample; (**b**) SA sample; (**c**) PA sample and (**d**) PSA sample tensile tested at 900 °C.

Figure 11a–d shows the fracture surfaces of the as-built sample and three heat-treated samples tensile tested at 900 °C. From the observation of Figure 11a,b, the fracture modes of quasi-cleavage intergranular cracking for the AB sample and SA sample are nearly the same. The molten tracks and checkboard pattern are still clearly seen due to the presence of columnar grains. It is known that rapid solidification starts at the edge of the molten pool and the solid-liquid interface growth changes along the molten pool, resulting in cellular structure and solute segregation. This leads to an increase in the area of substructure boundaries, creates more mechanically weak regions, and the transgranular crack propagation mode changes to intergranular mode [55]. It is thus believed that the substructure and columnar grain boundaries act as crack initiators and propagators at 900 °C which intercepts the lower elongation (~7%) in comparison to the SA sample. Although the quasi-cleavage intergranular mode is similar to that of the AB sample, the discrete nature of the carbide particles is able to prevent crack propagation in the SA sample, which leads to the higher elongation than that of the AB sample.

Regarding the PA and PSA samples, the fracture patterns are mainly rock-candy shaped intergranular fracture modes due to the conversion from cellular substructures and columnar grains to equiaxed grains arising from the recrystallization process. The cross-section morphologies also demonstrate that secondary cracks readily propagate along grain boundaries, which is detrimental to ductility. In contrast to the PSA sample, the continuous GB carbide particles in the PA sample lead to relatively higher strength and premature failure.

Numerous variables influence the tensile characteristics of a nickel-based superalloy strengthened with γ′, including the configuration of the γ′ phase (including its volume and size), the temperature at which the sample is tested, and the particle orientation, particularly in the case of specimens manufactured by AM. The experimental results above indicate that the transformation from inter- and trans-granular fracture modes, which exhibits a considerable elongation (>32%) at room temperature to single inter-(quasi) granular fracture mode, which experiences a ductility dip of over 50% at 900 °C is highly related to the change in deformation mechanism. At room temperature, dislocations only slip in the Ni matrix along the slip system of <110> {111} due to the absence of the γ′ phase and very fine carbide particles in the AB sample. The fracture surface exhibits a 45° inline tendency with the tensile axis. This inline angle is equivalent to the angle formed by the primary orientation of <100> and the slip system, as displayed in Figure 10a. Although the deformation mechanism of the SA sample is similar to that of the AB sample, the dislocations slipping in the {111} plane depend on the {111} plane direction of spherical γ′ particles, which induce cracks generating and propagating along {111} plane. Therefore, the relatively flat regions are more clearly seen in the macroscopic fracture surface. In the case of PA and PSA samples with random orientation of equiaxed grains, the tensile deformation usually follows Schmid’s law: sliding is initiated in a grain when the critical resolved shear stress (CRSS) is satisfied. The stress will be transferred to adjacent grains in the direction with the maximum Schmid factor [56]. In addition, parts of grains might rotate to maintain the sliding. As a result, the fracture surfaces of them look tortuous.

A decrease in both strength and ductility is observed at 900 °C due to the weakening of the γ matrix and γ′ precipitates and grain boundary instability. First, the cavitation, void nucleation and growth become the primary causes of intergranular cracking when the tensile stress is high enough to devastate the interlink between the γ matrix and carbide particles at grain boundaries at 900 °C. There are only very fine carbide particles in the AB sample. The micro-segregation and residual stress are presented at the columnar grain boundaries and substructure boundaries at which cracks propagate to form cleavage facets, as seen in the literature [57,58]. Following the heat treatments, the carbide phase has a striking influence on the tensile properties. The carbide phase is hard and brittle and not closely combined with the matrix. The dislocation pile-ups accumulate around the hard particles and a great number of dislocations are stacked in the matrix channels when deformation occurs. The more carbides there are, the more difficult the dislocation movement is [48,59]. As known from Figure 7 and Figure 9, the highest content of the carbide phase has a size much larger than that of the γ′ phase in the PA sample. The continuous or semi-continuous carbide particles in the PA sample prevent the dislocation’s movement across grain boundaries, resulting in stress concentration and the formation of micro-voids and cracks. Also, grain boundary triple junctions act as a stress concentration site resulting in reduced ductility. Therefore, the dispersive large size of carbide and small size of the γ′ phase lead to the higher strength of the grain than that of the grain boundary. In addition, the straight grain boundary sliding and premature instability happen more easily. The SA and PSA samples which are decorated with discontinuous carbide particles possess higher resistance to straight grain boundary sliding, thereby enhancing tensile strength and facilitating the movement of dislocations across grain boundaries [60]. The change in the deformation mechanism is another cause behind the ductility drop at 900 °C. According to several pieces of research [61,62,63], once the deformation mechanism changes, the ductility might decrease simultaneously. The size of the γ′ phase and deformation temperature usually affect the deformation mechanism. Dislocations shear the γ′ precipitates at low and intermediate temperatures (below 650 °C and 850 °C), whereas the Orowan bypass mechanism prevails at high temperatures (above 900 °C). As the temperature increases, the deformation activation energy decreases and the dislocation climbing mechanism is initiated arising from thermal activation, where the stress required does not reach the critical stress required for the dislocation cutting γ′ phase, leading to bypassing the γ′ precipitates [64]. Shin et al. [65] measured the different size distributions by tuning the cooling rate of solid solution treatment and calculated the critical size of the γ′ phase distinguishing dislocation shearing or bypassing. At 750 °C, more than 20% of elongation and a plastic behavior were achieved in the sample with which the size of the γ′ phase was less than the critical size. In our study, when loading at room temperature, the high-density dislocations slip in the Ni matrix, leading to the superior work hardening in the AB sample. The combination of inter- and trans-granular fracture modes in the aged samples indicates that the dislocations move through shearing of the γ′ precipitates in pairs of a/2<101> matrices [62,66]. Zhang et al. [28] obtained γ′ phases measuring approximately 50 nm on average. They found a combination of dislocation loops around the γ′ particles and dislocation shearing through the γ′ particles after loading at 900 °C. Based on their work, at 900 °C, there is a high probability that the deformation mechanisms in the PA and PSA samples are the strongly coupled dislocation shearing and Orowan bypass, resulting in a decrease in strength [67,68]. In addition, it is inferred from intergranular fracture mode at 900 °C that the strength stemming from the carbide pinning is weaker than that stemming from the CRSS within grains when loading at 900 °C. At last, the premature failure at grain boundaries at 900 °C brings about the “rock-candy” fractured surface. Therefore, we will further investigate the correlation between deformation mechanisms and fracture modes of the new heat-treated sample in future research.

## 5. Conclusions

In this study, the GH4099 superalloys produced by laser powder bed fusion (LPBF) were heat-treated using three strategies, in which their microstructural evolution and tensile properties at room temperature (RT) and 900 °C were systematically investigated. The main conclusions can be summarized as follows:

1. The as-built alloy exhibits the typical cellular/dendrite substructures within columnar grains with <100> crystal orientation along the building direction and without a γ′ phase. Very fine substructures and high-density entangled dislocations contribute to high RT ductility (approximately 51% elongation and 67% area reduction), but embrittlement occurs at 900 °C (typical cleavage facets).
2. Upon the standard solid solution heat treatment, the molten pool boundaries in the SA sample are eliminated but the columnar grains still remain. A small quantity of discrete M_23_C_6_ carbide particles are distributed along grain boundaries and original substructure boundaries. With increases in solid solution temperature, the transformation from columnar grains to equiaxed grains occurs due to the recrystallization process. The fine primary MC particles merge and grow up. Then, after heat treatment by the subsequent secondary solid solution, the discrete M_23_C_6_ carbide particles again precipitate along grain boundaries and original substructure boundaries according to the precipitation reaction MC + γ→M_23_C_6_ + γ′. Meanwhile, the large size of the cubic γ′ phase precipitates and grows. Following the aging process, the spherical γ′ phase uniformly disperses in the matrix among three different heat treatment samples while the size and volume fraction of the carbide phases in the three samples further increase.
3. The SA sample shows the highest RT yield strength due to the presence of remaining entangled dislocations. The PA and PSA samples show similar RT yield strength and tensile strength. The fracture behaviors of all three samples are a combination of intergranular (GB cracks) and transgranular (dimples) cracking. However, a clear ductility dip during load testing at 900 °C comes up in the AB sample and three heat-treated samples. The large carbide particles with grains and the semi-continuous carbide particles at grain boundaries significantly prevent the movement of dislocations, giving rise to high strength and low ductility. Conversely, the discontinuous M_23_C_6_ carbide particles at GBs in the PSA sample can effectively prohibit grain boundaries from sliding. It thus achieves relatively high elongation at 900 °C. However, there are no exceptions that their fracture modes are inter-(quasi)granular cracking. The change in deformation mechanisms from room temperature to 900 °C might be the main cause of plasticity-embrittlement transformation.

## Figures and Tables

**Table 1:** Chemical compositions of the GH4099 alloy powder (wt.%).

Cr	Co	W	Mo	Al	Ti	C	Fe	Si	Mn	O	N	Ni
18.08	6.68	5.91	4.00	2.18	1.4	0.053	0.70	0.062	0.014	0.0091	<0.001	Bal.

**Table 2:** Heat treatments for LPBF GH4099 superalloy.

Sample	Standard Solid Solution	Primary Solid Solution	Secondary Solid Solution	Aging
	1140 °C × 120 min	1205 °C × 120 min	1010 °C × 240 min	850 °C × 480 min
SA	√			√
PA		√		√
PSA		√	√	√

**Table 3:** Grain boundary misorientation (%) in the BD of SA, PA and PSA sample.

Sample	LAGB (2°~15°)	HAGB (>15°)	60° ± 5° (TB)
SA	41.2	58.8	0.4
PA	9.9	90.1	48.7
PSA	2.3	97.7	56.4

**Table 4:** Recrystallization mapping results (%) in the BD of SA, PA and PSA sample.

Sample	Recrystallized Grain	Substructure Grain	Deformed Grain
SA	2.85	22.58	74.57
PA	63.35	25.25	11.40
PSA	66.74	26.53	6.73

## Data Availability

Data are contained within the article.

## Supplementary Materials

The following supporting information can be downloaded at: https://www.mdpi.com/article/10.3390/ma17051084/s1, Figure S1: Microstructures of YOZ plane (a,b) and XOY plane (c,d). Figure S2: The inverse pole figure (IPF) and grain size distribution from EBSD of the AB sample (a) YOZ plane; (b) XOY plane. Figure S3: (a,b) TEM bright-field images of the AB sample; (**c**) TEM-EDS mapping results. References [11,52,57,69,70,71,72] are cited in the Supplementary Materials.
