# Supplementary material for: Influence of Various Heat Treatments on Microstructures and Mechanical Properties of GH4099 Superalloy Produced by Laser Powder Bed Fusion
Source: Materials (Basel). 2024 Feb 27;17(5):1084. doi: 10.3390/ma17051084 (PMC10934370; doi:10.3390/ma17051084)
Supplement: Supplementary file 1 [file materials-17-01084-s001.zip › materials-2846079-supplementary.pdf]

materials

Supplementary Information for:

Influence of Various Heat Treatments on Microstructures and Mechanical Properties of GH4099 Superalloy Produced by Laser Powder Bed Fusion

Jiahao Liu [1,2], Yonghui Wang [3], Wenqian Guo [3], Linshan Wang [1,3], Shaoming Zhang [1,2] and Qiang Hu [1,2,*]

[1] Industrial Research Institute for Metal Powder Material, China GRINM Group Co., Ltd., Beijing 101407, China; lewin068@163.com (J.L.); wls@grinm.com (L.W.); shaoming_zhang@126.com (S.Z.)
[2] General Research Institute for Nonferrous Metals, Beijing 100088, China
[3] GRINM Additive Manufacturing Technology Co., Ltd., Beijing 101407, China; wangyonghui80901@126.com (Y.W.); gwq812869@126.com (W.G.)
* Correspondence: Correspondence: huqiang@grinm.com

The optical micrographs (OM) and SEM images of YOZ plane and XOY plane of the as-built GH4099 superalloy are observed in Figure S1. The typical layer-by-layer scale-like molten pool morphology due to the Gaussian energy distribution of the laser spot is seen in the YOZ plane. A large number of primary columnar dendrites grow epitaxially along the building direction through the multiple-layer molten pools. The width of the molten pools is 90 μm-100 μm and the depth of the molten pools is 80 μm-100 μm, in which they mutually form great metallurgical bonding. The XOY plane morphology of the as-built alloy shows the molten tracks stacked in layers, overlapping each other laterally with the width of 100 μm-120 μm. The primary columnar dendrites and cellular structure of YOZ plane of AB sample from the high magnification SEM images can be observed. The primary columnar dendrites grow epitaxially along the building direction with an average dendrite arm spacing of 0.8 μm-1 μm. The primary dendrites growth direction is determined by preferred orientation of matrix and opposite to the maximum temperature gradient direction during the solidification process. The secondary dendrites arms are underdeveloped owing to the rapid cooling rate.

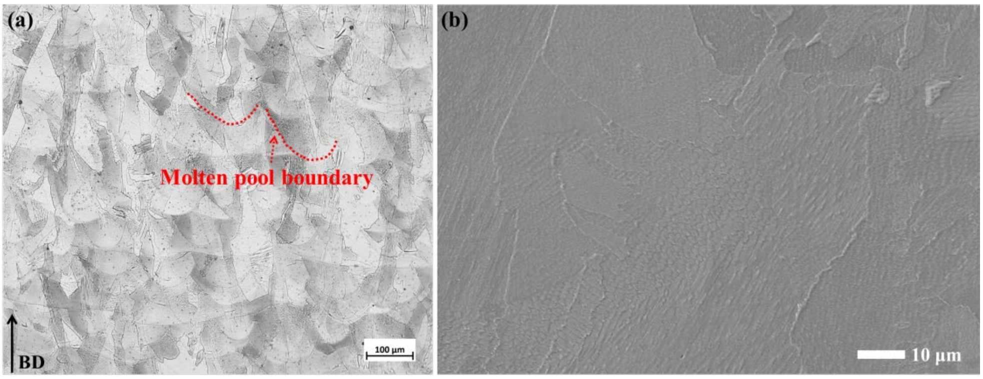

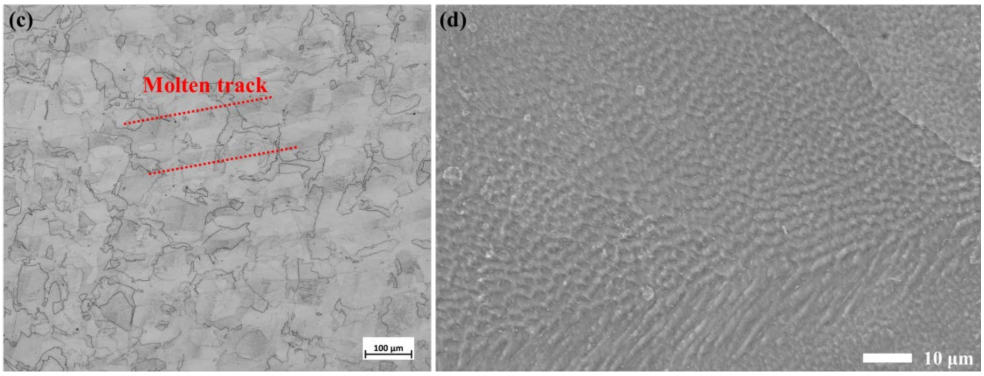

Figure S1. Microstructures of YOZ plane (**a**,**b**) and XOY plane (**c**,**d**).

To evaluate the grain orientations and morphologies of the AB sample, EBSD results are acquired from both the YOZ and XOY plane, as displayed in Figure S2. As the grains do not present round shape in cross-section plane, the measure of "equivalent circle diameter" has been considered based on grain area. Figure S2(a) depicts that alloy consists of elongated columnar grains along the building direction with the preferred orientation of <001>. On the other hand, XOY plane contains a chessboard-like grain pattern due to the scanning strategy (67° rotation angle between longitudinal and lateral row). The average grain size of YOZ plane is ~99.15 µm due to their columnar shape, whereas that of XOY plane is only ~51.31 µm. Also, very-fine dendritic structure within large columnar grains contributes to the increase of grain boundary area.

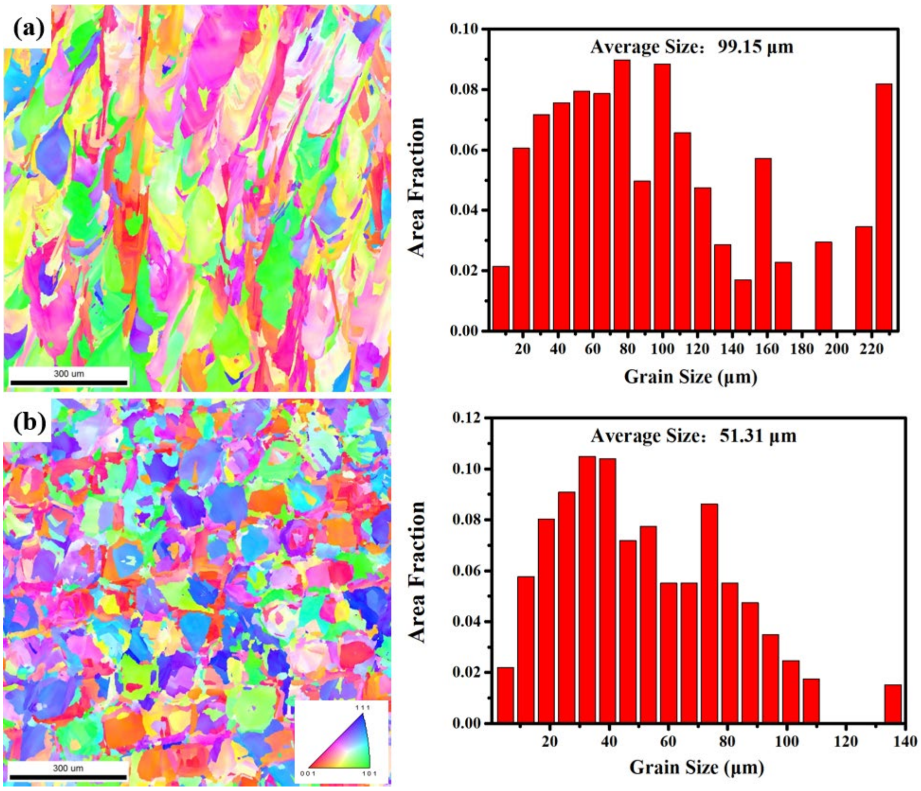

Figure S2. The inverse pole figure (IPF) and grain size distribution from EBSD of the AB sample (**a**) YOZ plane; (**b**) XOY plane.

From the TEM bright-field images, the high-density entangled dislocations can be observed at the cellular substructure boundaries, which reflects the high magnitude of residual stress in the AB sample, as displayed in Figure S3 (**a**,**b**). No primary γ' phase is seen because the content (3.58 wt.%) of Al+Ti is not much high enough to precipitate under

such rapid cooling rate, even if the repeated thermal cycles which essentially provide an "aging" condition. Additionally, a host of carbide particles distribute in the alloy. According to the TEM-EDS mapping result, as illustrated by Figure S3 (c), Ti, Mo, C incline to micro-segregate together leading to the formation of carbide particles whose size is less than 30 nm and others basically evenly distribute, which determines that MC carbide forms during the solidification. MC carbide generally forms during the solidification of superalloys, as described by many work [1–3]. The MC carbide particles in the AB sample has such a fine size as a result of the rapid cooling rate

Such microstructural characteristics of AB sample are basically consistent with other LPBF superalloy researches [4–7]. Therefore, the microstructural evolutions after solid solution treatment and aging treatment are mainly discussed in the manuscript.

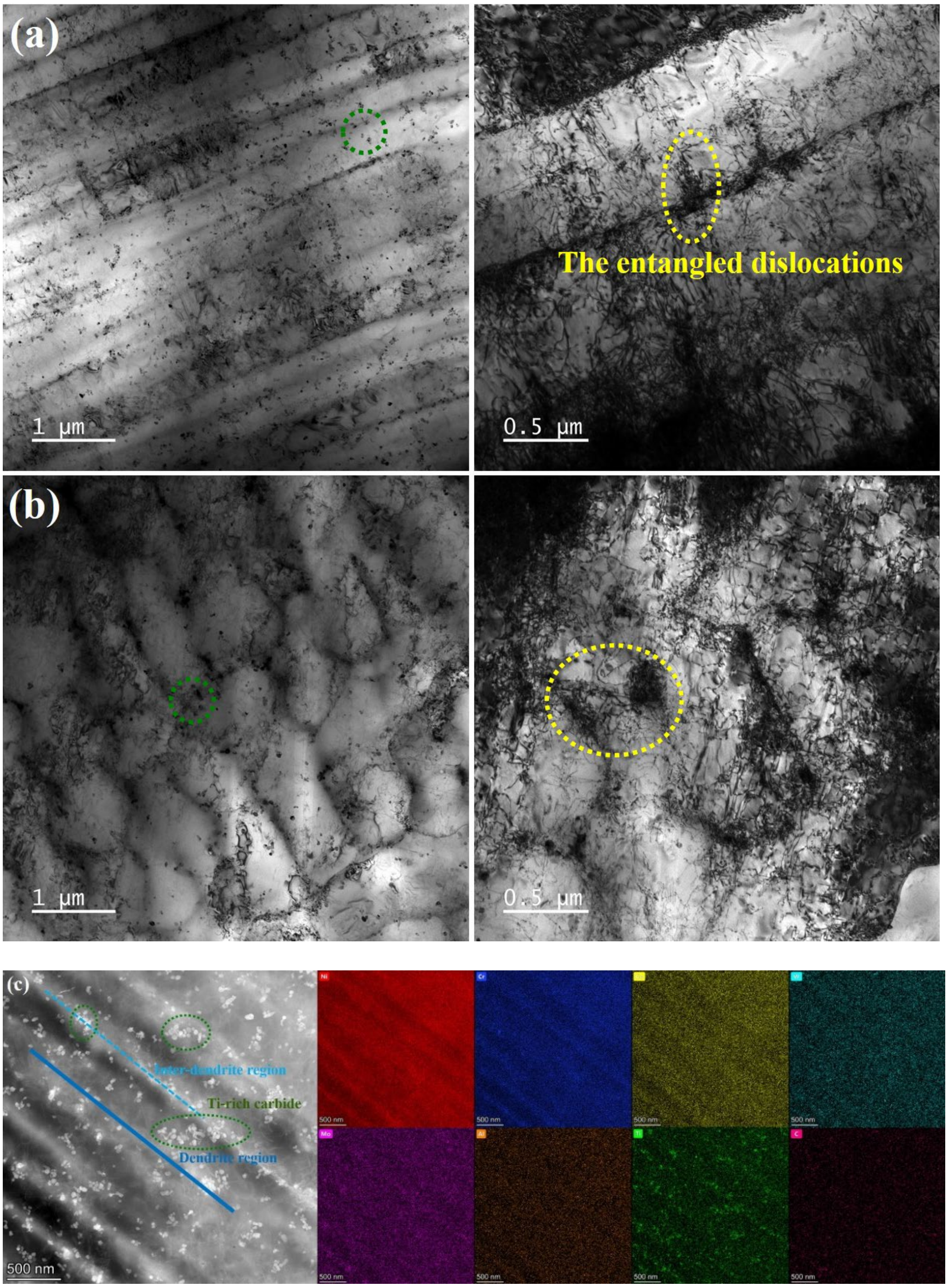

Figure S3. (**a**,**b**) TEM bright-field images of the AB sample; (**c**) TEM-EDS mapping results.

References

1. Brittan, A.; Mahaffey, J.; Anderson, M. The performance of Haynes 282 and its weld in supercritical CO_2. *Mater. Sci. Eng. A* **2019**, *759*, 770–777.
2. Zhang, H. L.; Wang, Y. K.; Vecchis, R. R. D.; Xiong, W. Evolution of carbide precipitates in Haynes® 282 superalloy processed by wire arc additive manufacturing. *J. Mater. Proc. Tech.* **2022**, *305*, 117597.
3. Jiang, H.; Xiang, X. M.; Dong, J. X. The morphology and characteristics evolution of MC carbide during homogenization in hard-to-deform superalloy GH4975. *J. Alloy. Compd.* **2022**, *929*, 167086.
4. Kangazian, J.; Shamanian, M.; Kermanpur, A.; Foroozmehr, E.; Badrossamay, M. Investigation of microstructure-tensile behaviour relationship in Hastelloy X Ni-based superalloy processed by laser powder-bed fusion: Insights into the elevated temperature ductility loss. *Mater. Sci. Eng. A* **2021**, *823*, 141742.
5. Zhao, J. R.; Hung, F. Y.; Lui, T. S. Microstructure and tensile fracture behavior of three-stage heat treated Inconel 718 alloy produced via laser powder bed fusion process. *J. Mater. Res. Tech.* **2020**, *9*, 3357–3367.
6. Hao, Z. B.; Tian T.; Peng, S. Q.; Ge, C. C.; Li, X. G.; Jia, C. L.; Guo, C.; Zhu, Q. Effect of Heat Treatment on Microstructure and Properties of FGH4096M Superalloy Processed by Selective Laser Melting. *Metals Mater. Int.* **2020**, *26*, 1270–1285.
7. Teng, Q.; Li, S.; Wei, Q.; Shi, Y. Investigation on the influence of heat treatment on Inconel 718 fabricated by selective laser melting: Microstructure and high temperature. *J. Manuf. Proc.* **2021**, *61*, 35–45.
